# Metabolic memory in the kidney: how lactate and lactylation drive the path from acute injury to chronic disease

**DOI:** 10.1080/0886022X.2026.2671466

**Published:** 2026-05-20

**Authors:** Xinyao Zhang, Qing Dai, Huiling Feng

**Affiliations:** Hunan University of Chinese Medicine, Changsha, China

**Keywords:** Lactate, lactylation, acute kidney injury, chronic kidney disease

## Abstract

Acute kidney injury is a primary risk factor for the development of chronic kidney disease, however, the molecular mechanisms driving this pathological progression remain to be elucidated. Recently, emerging evidence indicates that lactate, a key metabolite, and its novel derivative post-translational modification, lysine lactylation, play a central role in the process of kidney injury and repair. This review systematically integrates the latest research on the lactate-lactylation axis in the transition from acute kidney injury to chronic kidney disease. We focus on four core maladaptive repair processes: mitochondrial dysfunction/redox stress, metabolic reprogramming, inflammation/immune remodeling, and fibrosis/cell state transitions, and provide an in-depth discussion of how lactylation, acting as a critical epigenetic switch, translates upstream metabolic disturbances into persistent cellular dysfunction and tissue damage. This article not only analyzes the specific molecular mechanisms of lactylation in each pathological process but also highlights its role as a central hub that couples these elements into a self-amplifying vicious cycle.

## 1. Introduction

Acute kidney injury (AKI) is a clinical syndrome defined by a rapid decline in renal function, which leads to the accumulation of metabolic waste products throughout the body [1]. Its primary clinical manifestations include disturbances in water, electrolyte, and acid-base balance, as well as azotemia, often accompanied by oliguria or anuria [2]. Epidemiological studies indicate that AKI affects approximately 10%–15% of hospitalized patients, with a mortality rate of 10%–20%. The incidence of AKI in intensive care unit (ICU) patients can exceed 50% [3–5]. Crucially, patients who experience AKI have an eight-fold higher risk of developing chronic kidney disease (CKD) compared to those without AKI, posing a severe threat to human health and imposing a substantial medical burden [6]. However, the mechanisms governing the progression from AKI to CKD remain largely unclear. Previous studies have implicated several factors in the AKI-to-CKD transition, including the expression of cytokines like hypoxia-inducible factors and transforming growth factors, as well as macrophage polarization and histone modifications [7–9]. Despite this knowledge, interventions targeting these mechanisms have not effectively delayed the onset of CKD. Therefore, a deeper exploration of the underlying drivers of the AKI-to-CKD transition is essential.

The discovery of lactic acid can be traced back to the late eighteenth century, when Swedish chemist Carl Wilhelm Scheele first isolated lactic acid from a sample of yogurt in 1780 [10]. Initially, lactate was considered merely a metabolic byproduct of cellular glycolysis under anaerobic conditions. This view began to shift in 1921, when Otto Warburg observed that tumor cells preferentially produce lactate through glycolysis even in the presence of ample oxygen—a phenomenon later termed the Warburg effect [11]. The traditional view of lactate as a metabolic waste product was overturned in the 1980s by George Brooks, who proposed the lactate shuttle theory, demonstrating that lactate is not merely an end product but also a key intermediate in glucose metabolism that actively participates in energy metabolism [12]. In addition to this, lactate has also been demonstrated in subsequent studies to function as a signaling molecule involved in signal transduction processes such as angiogenesis, tumor metastasis, and immune regulation [13]. In 2019, a pivotal discovery by Zhang et al. established that lactate can act as a novel epigenetic precursor, inducing a post-translational modifications (PTMs) on histones known as lactylation [14]. This finding has opened new avenues for research into lactate-mediated signaling. This discovery not only highlights lactate’s ability as a metabolite to alter protein function but also reveals the intricate relationship between metabolites and gene regulation. In subsequent studies, lactylation has been found to be involved in coordinating various pathophysiological processes, including tumorigenesis, immune responses, and inflammatory responses [15]. Furthermore, studies on the role of lactylation in the pathogenesis of cardiovascular diseases and neurological disorders have been reported successively [16,17]. In the context of kidney disease, lactylation has been found to promote the progression from AKI to CKD by facilitating inflammation and fibrosis [18]. This body of evidence underscores the broad role of lactate and lactylation in disease and provides a strong rationale for their involvement in the pathogenesis of the AKI-to-CKD transition.

In a recently published article, Hou et al. also shed light on the emerging field of renal lactylation [19]. They provided an extensive and systematic summary of the roles of lactate and lactylation in various diseases. However, the role and specific mechanisms of the lactate–lactylation axis in the transition from AKI to CKD remain unclear. For AKI, mitochondrial dysfunction, metabolic reprogramming, inflammatory injury, and fibrotic responses are all involved in the maladaptive repair process [20–22]. Therefore, we believe that these related pathological processes may constitute the core of maladaptive repair in AKI. Lactylation, serving as a direct link between lactate and epigenetic modifications as well as altered metabolic functions, may constitute the molecular basis of the aforementioned maladaptive repair processes, thereby driving the progression to CKD.

Therefore, this review provides a more detailed discussion of the impact of the lactate-lactylation axis in the transition from AKI to CKD by further analyzing relevant literature. Moreover, by excavating the underlying mechanisms through which this axis drives AKI-CKD progression, it aims to elucidate the role of lactate and lactylation in the advancement of AKI to CKD, offering clear and rational guidance for understanding how this critical metabolic-epigenetic axis contributes to the continuum of kidney disease progression.

## 2. The relationship between AKI and CKD

Historically, AKI was considered a self-limiting disease with a favorable long-term prognosis once renal function returned to baseline [23]. However, accumulating research has revealed that the effects of AKI can persist long-term and may accelerate the progression to CKD. For example, a retrospective study following 3809 individuals found that the incidence of subsequent CKD was significantly higher in patients with a history of AKI compared to those without (15% vs. 3%, respectively) [24]. Establishing a direct causal link between AKI and CKD is challenging due to confounding factors such as shared risk factors, variability in patient follow-up, and an incomplete understanding of CKD development [25]. Nevertheless, a past meta-analysis of relevant studies indicates that, compared to patients without AKI, those who experience an AKI episode face a significantly higher risk of developing CKD (pooled adjusted hazard ratio (HR) 8.8) and mortality (pooled adjusted HR 2.0). When AKI is categorized into mild, moderate, and severe grades, the risk of developing CKD increases in a graded manner with the severity of AKI [6]. Furthermore, a recent meta-analysis provides additional support for this perspective. By analyzing 165,715 medium-to-high quality studies related to AKI, researchers found that patients with AKI have a higher risk of developing CKD (HR 2.36), CKD progression (HR 1.83), kidney failure (KF) (HR 2.64), and major adverse kidney events (MAKE) (odds ratio (OR) 2.77) compared to those without AKI. Importantly, this risk remains significant even when AKI is at stage 1 (HR 1.49) [26]. In addition, evidence from some past animal experiments also supports the progression from AKI to CKD. By modeling in rats, researchers observed that AKI can lead to increased levels of transforming growth factor and angiotensin II in the rats, and these cellular molecules may play an important role in the subsequent development of CKD [27,28]. In some recent studies, it has also been found that fibroblast growth factor 2 (FGF2) produced by renal tubular cells *via* autophagy after AKI can contribute to the subsequent progression of CKD [29]. With the continuous advancement of experimental research, the pathophysiological mechanisms underlying the transition from AKI to CKD have become increasingly clear. Relevant reviews summarize that processes such as tubular epithelial cells (TECs) injury and apoptosis, ferroptosis, inflammation and immune responses, as well as fibrosis play crucial roles in this transition [30]. In addition, certain studies on metabolism and bioenergetics also provide important insights into the relationship between AKI and CKD. For example, the post-ischemic inactivation of prolyl hydroxylase 1–3 (PHD1–3) exacerbates the transition from AKI to CKD by promoting maladaptive renal repair through the induction of glycolysis [31]. Meanwhile, another study demonstrated that inhibiting sodium-glucose cotransporter 2 (SGLT2) reversed mitochondrial dysfunction, thereby preventing maladaptive repair after AKI and slowing its progression to CKD [32]. Although the precise mechanisms of the AKI-to-CKD transition remain to be fully elucidated, evidence from clinical studies, meta-analyses, and animal experiments consistently points to AKI as a significant risk factor for the development of CKD.

## 3. Biological characteristics of lactate and lactylation in the kidney

In nature, lactate exists in two configurations: D-type and L-type. However, the human body contains only the L-type [33,34]. As a product of cellular glycolysis, lactate can be produced by most tissue cells in the human body, with muscle cells being the most notable producers [33,35]. For the human body, under normal physiological conditions, the concentration of lactic acid is maintained at approximately 1–2 mM [36]. However, under certain specific circumstances, for example, when the human body undergoes vigorous exercise or suffers from certain diseases (such as diabetes, sepsis, shock, cancer, etc.), the lactate concentration can increase significantly [36–38]. This abnormal lactate concentration may lead to body damage, consequently making the lactate clearance function in the human body particularly important. As the largest metabolic organ, the liver can clear approximately 70% of the lactate throughout the body by producing glucose through glyconeogenesis [35]. Additionally, some lactate can be directly utilized for energy by cardiac muscle cells [39]. Not only that, the filtration function of the kidneys can also directly eliminate a portion of lactate from the body [35]. It is worth noting that the renal gluconeogenesis also plays a significant role in lactate clearance.

### 3.1. Lactate metabolism in the kidney

Studies have shown that lactate can serve as an important precursor for renal gluconeogenesis and participate in the synthesis of endogenous glucose. When liver function is impaired, the inhibition of the tricarboxylic acid (TCA) cycle in hepatocytes leads to a significant increase in lactate levels, which in turn further enhances compensatory gluconeogenesis in the kidneys [40]. In normal individuals, the kidneys play a critical role in systemic gluconeogenesis after an overnight fast (14–16 h), contributing approximately 40% of total endogenous glucose production. Moreover, renal gluconeogenesis exhibits significant regional specificity, being primarily localized in the cortical proximal tubular cells (PTCs) of the kidney [41]. This is intricately linked to the distinct energy metabolism patterns resulting from regional differences in blood supply within the kidneys. In the renal medulla, where blood supply is relatively insufficient, glucose metabolism primarily occurs anaerobically, leading to substantial accumulation of lactate in this area. In contrast, the proximal tubules located in the renal cortex rely mainly on aerobic respiration for energy metabolism due to adequate blood supply [42]. Therefore, the lactate produced in the medullary region serves as an efficient source of gluconeogenic substrate for PTCs in the cortex, thereby establishing a unique corticomedullary glucose-lactate cycle within the kidney [43]. This metabolic mechanism not only reflects the regional specificity of the kidneys in glyconeogenesis but also reveals the metabolic synergy between the cortex and medulla.

### 3.2. Lactylation modification: a novel bridge transducing metabolic signals into functional regulation

**Figure 1.**
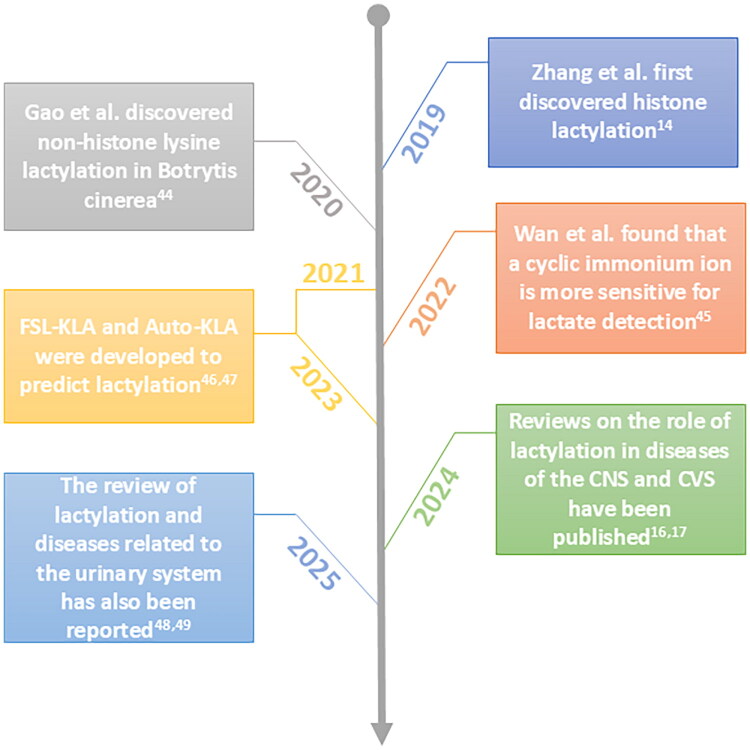
Timeline of lactylation research development. This timeline outlines key milestones in lactylation research, tracking its progression from initial discovery to the current understanding of its mechanisms and implications across biological systems. It highlights major events in the discovery of lactylation, its identified locations, detection methods, site prediction tools, and its documented roles in various physiological and pathological systems, providing a clearer perspective on this novel molecular mechanism.

**Figure 2.**
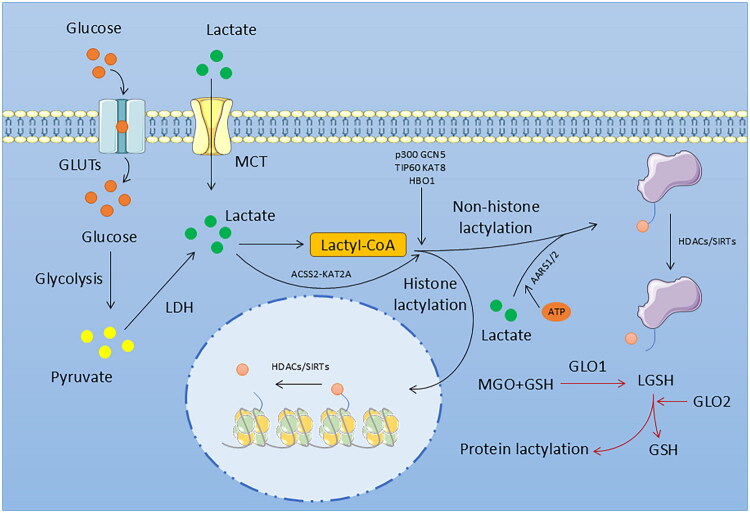
The mechanism of lactylation in cells. Exogenous lactate enters the cell *via* MCT, while endogenous lactate is generated through the glycolytic pathway. Lactate from both sources can be catalyzed to form lactyl-coenzyme A, and then lactylation of proteins is mediated by various writer proteins. MCT: Monocarboxylate transporter; GLUTs: Glucose Transporters; LDH: Lactate Dehydrogenase; HDACs: Histone Deacetylases; SIRTs: Sirtuins; AARS1/2: Alanyl-tRNA synthetase 1/2; MGO: Methylglyoxal; GSH: Glutathione; GLO1/2: Glyoxalase 1/2; LGSH: Lactoylglutathione.

Since the discovery of lactate, our understanding of it has been evolving gradually. Lactate is not only a substrate for energy metabolism, but also a key molecule involved in the regulation of body signaling. In 2019, Zhang et al. discovered that lactate possesses a new molecular function—lactylation [14]. This also sets the stage for its subsequent related research (Figure 1). From histone sites to non-histone sites, and evolving detection methods, research on lactylation sites has encompassed various aspects, including humans, animals, plants, and microorganisms [44–47]. Beyond research focused on specific lactylation sites, studies investigating the role of lactylation in the occurrence and progression of diseases across various organ systems—such as the cardiovascular, nervous, and urinary systems—are also continually emerging [16,17,48,49]. Not only that, the mechanisms regulating the initiation and termination of lactylation are gradually becoming clear. These enzymes are commonly referred to as the ‘writers,’ ‘readers,’ and ‘erasers’ of lactylation (Figure 2) [50]. In the initial study, p300 was first discovered to catalyze the conjugation between the lactate group of lactoyl-CoA and specific lysine residues [14]. Next, several other histone acetyltransferases (HATs), including GCN5, TIP60, KAT8, and HBO1, were subsequently confirmed to participate in histone lactylation writing [51–54]. Furthermore, alanyl-tRNA synthetase AARS1/2 can also undergo lactylation modification in the presence of ATP [55,56]. Not only that, but the latest research also shows that under conditions where it acts as a lactyl-CoA synthetase, acetyl-CoA synthetase 2 (ACSS2) can couple with KAT2A to function as a lactate transferase, thereby mediating histone lactylation [57]. However, in addition to enzyme-catalyzed induction of lactylation, there is in fact a non-enzyme-mediated form of lactylation in the body—acyl transfer involving lactoylglutathione for non-enzymatic lactylation [58]. Although some progress has been made in the study of lactylation writing proteins, research on lactylation erasing proteins remains relatively limited. Current studies indicate that Histone Deacetylase (HDAC)1–3 in the HDAC family and Sirtuin (SIRT)1–3 in the SIRT family play certain roles in the delactylation process of histones [59]. Furthermore, in subsequent studies, SIRT3 was also found to inhibit the development of hepatocellular carcinoma by delactylating the non-histone protein cyclin E2 (CCNE2) [60]. In addition to the presence of enzymes related to lactylation in the human body, researchers have also detected YiaC and CobB in Escherichia coli, which function as writer and eraser proteins for lactylation, respectively, playing important roles in the metabolic processes of prokaryotic organisms [61]. Although some related mechanisms have been understood, they are not yet perfect. Continuous research on these enzymes is of great importance for inhibiting the occurrence and development of diseases in the future and for maintaining the health of the organism. For the kidneys, particularly in previous related studies on the progression from AKI to CKD, PTMs, as a crucial form of epigenetic modification, have garnered significant attention in this regard. Histone methylation, acetylation, phosphorylation, and ubiquitination have been widely demonstrated to promote the transition from AKI to CKD by facilitating inflammation and fibrosis [9]. In contrast, lactylation, as a newly discovered post-translational modification, requires further exploration regarding its role in kidney diseases, especially in the progression from AKI to CKD.

## 4. The dual roles of lactate and lactylation

While lactate has historically been viewed as a detrimental metabolic waste product, recent literature reveals a highly context-dependent dual role in kidney disease. The effects of lactate and lactylation depend heavily on the timing of the injury, the specific cellular compartment, and the cell type involved.

Timing (Injury vs. Maladaptive Repair): For AKI, mitochondrial damage in PTCs during the disease leads to the inhibition of fatty acid β-oxidation (FAO), and to sustain normal energy supply, PTCs protect the kidneys by enhancing the glycolytic pathway to produce lactate [62]. Furthermore, a study on folic acid (FA)-induced AKI has also revealed that lactate promotes macrophage polarization and fibroblast activation, thereby facilitating the repair of kidney injury [63]. Furthermore, for early AKI, the proliferation of renal TECs is crucial for renal function recovery, and the lactylation of the specific transcription factor c-Myc promotes this process, thereby aiding in the restoration of ischemia-reperfusion (I/R) injury [64]. However, when glycolysis is persistently enhanced and lactate continuously accumulates in cells, the kidneys will enter a stage of maladaptive repair, and lactate will also transform from a protective energy source into a pro-fibrotic and pro-inflammatory epigenetic signal [31]. This indicates that lactate, as a metabolic product of the body, plays a dual role in either promoting repair or exacerbating damage at different stages of disease progression.

Compartmental and Cell-Specific Effects: As mentioned previously, depending on the environment, the choice of energy metabolism also varies. In the kidney, lactate forms a unique glucose-lactate cycle between the renal cortex and medulla. Lactate plays an important role as a substrate for energy metabolism in the cortex. Moreover, the physiological effects of lactate are strictly compartmentalized both inside and outside the cells. Within cells, lactate accumulation in TECs drives epigenetic alterations (such as H3K18la), which promote the sustained progression of inflammation and lead to apoptosis [65]. Extracellularly, lactate transported outside the cells alters the renal microenvironment and, as a signaling molecule, promotes M2 macrophage polarization, thereby driving immune remodeling and fibrosis progression [66].

Evaluation of Current Evidence: A critical assessment of the existing body of evidence is essential. Current foundational knowledge largely relies on correlational observations, such as noting that global lactylation levels increase following injury alongside rising lactate concentrations [14]. Although research on causal perturbation interventions—such as genetic or pharmacological manipulation of glycolysis‑related enzymes (e.g., hexokinase (HK), pyruvate kinase M2 (PKM2)) or specific lactylation‑modifying enzymes/de‑modifying enzymes (e.g., p300, SIRT3)—has begun to emerge, these studies have thus far remained largely confined to preclinical mouse *in vivo* models (e.g., cecal ligation and puncture, unilateral ureteral obstruction) or *in vitro* bulk cell analyses [66–68]. Evidence from human clinical trials or single-cell-specific analyses that can clearly delineate the precise trajectory of lactylation in the human fibrotic microenvironment (fibroblasts/pericytes) remains limited. Future studies should prioritize the use of cell-specific knockout techniques to clearly distinguish between the autonomous and non-autonomous roles of lactylation in the kidney.

## 5. The mechanism of maladaptive repair in the transition from AKI to CKD

A considerable number of studies on lactylation in relation to AKI or CKD have been published (Table 1). However, the findings in most articles remain confined to a single disease context, and the specific mechanisms underlying the transition from AKI to CKD are still not well understood. For patients with AKI, when AKI occurs, the disease places renal cells in a pathological state, inevitably leading to corresponding metabolic disturbances. This ultimately results in the excessive accumulation of lactate in the kidneys. During the subsequent recovery phase of AKI, mitochondrial dysfunction/oxidative stress, metabolic reprogramming, inflammation and immune remodeling, as well as fibrosis and cellular state transitions, significantly promote the occurrence of adverse repair. This partially overlaps with the mechanisms underlying the progression of CKD. Therefore, as a key metabolite in this process, lactate may participate in the central mechanisms of these maladaptive repair processes by establishing a lactate-lactylation axis—a critical epigenetic and metabolic functional mechanism—thereby promoting the progression of AKI to CKD.

**Table 1.** Context-dependent mechanisms of the lactate-lactylation axis in kidney disease.

Disease model	Implicated cell type/compartment	Lactate axis component & target site	Major phenotype	Evidence type	References
Sepsis (CLP)	HK-2	Lactylation machinery: Fis1 K20	Mitochondrial dysfunction (Injury)	Perturbation (*in vivo*/*in vitro*)	[67]
Sepsis (CLP)	HK-2	Lactylation machinery: H3K18 and Ezrin K263	Inflammation/apoptosis	Perturbation (*in vivo*/*in vitro*)	[65]
Sepsis (CLP)	Macrophages	Lactylation machinery: HMGB1	NET formation	Perturbation (*in vivo*/*in vitro*)	[69]
I/R-AKI	HK-2	Lactylation machinery: H3K18	Enhanced glycolysis	Perturbation (*in vivo*/*in vitro*)	[70]
I/R-AKI	TEC	Lactylation machinery: c-Myc	TEC proliferation	Perturbation (*in vivo*/*in vitro*)	[64]
CDDP/MA-AKI	HK-2	Lactylation machinery: ALDH2 K52	Mitochondrial dysfunction (Injury)	Perturbation (*in vivo*/*in vitro*)	[71]
Lactate-AKI	HK-2	Lactylation machinery: HMGB1	NET formation	Perturbation (*in vivo*/*in vitro*)	[72]
DN (db/db)	HK-2/mTEC	Lactylation machinery: H3K14	EMT	Perturbation (*in vivo*/*in vitro*)	[73]
DN (STZ)	MPC	Lactylation machinery: Histones	EMT of podocytes	Perturbation (*in vivo*/*in vitro*)	[74]
DN (db/db)	HK-2	Lactylation machinery: ACSF2 K182	Mitochondrial dysfunction	Associative (*in vivo*/*in vitro*)	[75]
DN (STZ)	mRTEC	Lactylation machinery: H3	Fibrosis	Perturbation (*in vivo*/*in vitro*)	[76]
DN (STZ)	GEnCs	Lactylation machinery: H3K18	Endomt/Fibrosis	Perturbation (*in vivo*/*in vitro*)	[77]
DN (STZ)	HK-2	Lactylation machinery: TRIM65 K206	Enhanced glycolysis	Perturbation (*in vivo*/*in vitro*)	[78]
DN (STZ)	HPCs	Lactylation machinery: LARS1 K970	Inhibition of podocyte autophagy	Perturbation (*in vivo*/*in vitro*)	[79]
LN (PBMC immune reconstruction)	MC/HEK293	Lactylation machinery: PBX1 K40	Proliferation of MC	Perturbation (*in vivo*/*in vitro*)	[80]
UUO-CKD	HK-2	Lactylation machinery: H4K12	M2 polarization	Perturbation (*in vivo*/*in vitro*)	[66]
CDDP-CKD	BUMPT	Lactylation machinery: H3K18	Fibrosis	Perturbation (*in vivo*/*in vitro*)	[81]

CLP: Cecal Ligation and Puncture; HK-2: Human Kidney-2 cells; Fis1: Mitochondrial fission 1 protein; H3K18: Histone H3 lysine 18; Ezrin: Ezrin protein; HMGB1: High Mobility Group Box 1; NET: Neutrophil Extracellular Trap; I/R-AKI: Ischemia/Reperfusion-Induced Acute Kidney Injury; TEC: Tubular Epithelial Cells; c-Myc: Cellular Myelocytomatosis oncogene; CDDP/MA-AKI: Cisplatin/Mannitol-Induced Acute Kidney Injury; ALDH2: Aldehyde Dehydrogenase 2; Lactate-AKI: Lactate-Induced Acute Kidney Injury; DN: Diabetic Nephropathy; db/db: Diabetic (db/db) mouse model; mTEC: Mouse Tubular Epithelial Cells; H3K14: Histone H3 lysine 14; STZ: Streptozotocin; MPC: Mouse Podocyte Cells; ACSF2: Acyl-CoA Synthetase Family Member 2; mRTEC: Mouse Renal Tubular Epithelial Cells; H3: Histone H3; GEnCs: Glomerular Endothelial Cells; TRIM65: Tripartite Motif Containing 65; HPCs: Human Podocyte Cells; LARS1: Leucyl-tRNA Synthetase 1; LN: Lupus Nephritis; PBMC: Peripheral Blood Mononuclear Cells; MC: Mesangial Cells; HEK293: Human Embryonic Kidney 293 cells; PBX1: Pre-B-Cell Leukemia Homeobox 1; UUO-CKD: Unilateral Ureteral Obstruction-Induced Chronic Kidney Disease; M2: M2 macrophage phenotype; H4K12: Histone H4 lysine 12; CDDP-CKD: Cisplatin-Induced Chronic Kidney Disease; BUMPT: Boston University Mouse Proximal Tubule cell line.

### 5.1. Mitochondrial dysfunction and oxidative stress

As the key site of cellular energy metabolism, the proper functioning of mitochondria is crucial for the vitality of tissues and organs in the body. Disruption of mitochondrial function at varying levels can occur by affecting mechanisms such as mitochondrial DNA repair, dynamics, autophagy, and biogenesis [20]. Persistent mitochondrial dysfunction can disrupt the balance of mitochondrial dynamics and energetics, thereby impairing normal renal function. This plays a significant role in the onset and progression of kidney diseases such as AKI and Diabetic nephropathy (DN) [82]. Ischemia, hypoxia, and toxic substances are direct factors causing mitochondrial damage [83]. During the occurrence of AKI, mitochondrial dysfunction typically leads to the excessive production and accumulation of reactive oxygen species (ROS), and the surplus ROS further exacerbates mitochondrial dysfunction. Meanwhile, mitochondrial dysfunction also accelerates the release of renal pro-inflammatory factors (such as IL-18 and IL-1β) by releasing harmful substances such as ROS, DNA, and cardiolipin. This, in turn, aggravates inflammation and fibrosis, promotes renal cell apoptosis, and inhibits tissue repair [84]. This largely contributes to the occurrence of CKD. In the related studies on AKI and CKD, lactate and lactylation have also been shown to significantly impact mitochondrial function. For instance, in studies related to sepsis-induced acute kidney injury (SAKI), Fis1 K20la promotes mitochondrial fission, ROS production, and mitochondrial apoptosis, ultimately exacerbating the progression of AKI [67]. In Cisplatin (CDDP)-AKI, ALDH2 K52la can promote mitochondrial dysfunction by facilitating the ubiquitin-proteasome degradation of PHB2 [71]. In summary, lactylation modification has been demonstrated to act on key mitochondrial proteins (such as Fis1 and ALDH2) during the progression of AKI, providing a direct molecular mechanism through which metabolic stress (lactate accumulation) translates into functional mitochondrial damage. Meanwhile, in DN, the high expression of acyl-CoA synthetase family member 2 (ACSF2) is accompanied by lactylation at the K182 site, which further aggravates mitochondrial dysfunction [75]. It is thus evident that these associated molecular mechanisms are crucial for understanding the persistent injury and impaired adaptive repair involved in the transition from AKI to CKD.

### 5.2. Metabolic reprogramming

As the key site for the kidney to perform its physiological functions, the normal energy metabolism of TECs is the foundation of renal function. When AKI or CKD occurs in the kidney, due to the highly active energy metabolism of TECs (particularly PTCs), significant alterations in their energy metabolism often take place to maintain normal energy supply and renal function after cellular injury. In AKI, such metabolic changes typically involve multiple contributing factors (including hypoxia, mitochondrial dysfunction, and mTOR/AMPK signaling pathway imbalances). For PTCs, their primary energy production mechanism shifts from FAO to glycolysis. This metabolic reprogramming ultimately plays an important role in the transition from AKI to CKD through lipid accumulation, inflammatory responses, and fibrotic reactions [21]. The enhanced glycolytic response further leads to excessive production and accumulation of lactate, making it particularly crucial to focus on the impact of lactate during disease progression. Previous studies (including SAKI, I/R-AKI, and DN) have demonstrated that increased glycolysis generates surplus lactate, which exacerbates the deterioration of AKI or CKD, with lactylation playing a key role in this process. Specifically, following the onset of AKI, sustained metabolic reprogramming (enhanced glycolysis) results in prolonged lactate accumulation. This persistent lactate signaling, through lactylation modifications (such as H3K18la), promotes the sustained activation of pro-inflammatory genes (such as those in the NF-κB pathway), thereby inhibiting the normal resolution of inflammation and initiating a fibrotic program. This mechanism may represent a critical pathophysiological node in the transition from AKI to CKD. Of course, further research is needed to expand on the underlying connection mechanisms. Regarding lactylation, whether it can serve as direct evidence of metabolic reprogramming warrants attention. A current I/R-related AKI experiment has demonstrated the enrichment of H3K18la at the HK2 promoter region, which subsequently leads to the upregulation of HK2 and enhances the glycolytic response [70]. Similarly, a study on DN has shown that lactate can contribute to the progression of DN by promoting ferroptosis and glycolysis through inducing lactylation at K206 of TRIM65 in TECs [78]. However, although direct evidence remains limited at present, it is already established that lactate and lactylation can induce mitochondrial dysfunction, which in turn exacerbates metabolic reprogramming and leads to sustained elevation of lactate levels. Consequently, this indirect regulatory impact warrants considerable attention. It thus becomes evident that lactate, lactylation, mitochondrial dysfunction, and metabolic reprogramming form a mutually reinforcing, cyclical relationship. Such a vicious cycle collectively plays a significant role in driving disease progression.

### 5.3. Inflammation and immune remodeling

Inflammation and renal fibrosis, as further consequences of mitochondrial dysfunction and metabolic reprogramming, are key contributors to the transition from AKI to CKD [21]. AKI is often accompanied by a strong inflammatory response, and this sustained and recurrent inflammation is one of the important reasons for the progression of AKI to CKD [85]. In a study on ischemic AKI, researchers observed that the inactivation of PHD1–3 in endothelial cells led to impaired kidney repair, enhanced glycolysis, and the occurrence of pro-inflammatory responses. Among these, the fibrotic response could be attenuated by inhibiting monocarboxylate transporter (MCT)4, thereby reducing lactate production through weakened glycolysis [31]. Although the inhibition of MCT4 has also been shown to alleviate inflammatory responses, its relationship with glycolysis and lactate remains to be further confirmed. However, it is certain that the reduction of inflammation and fibrosis plays a role in mitigating the progression of AKI to CKD. Persistently enhanced glycolysis in cells has been shown to promote the occurrence and progression of CKD. The role played by 6-phosphofructo-2-kinase/fructose-2,6-bisphosphatase 3 (PFKFB3), one of the key enzymes in glycolysis, in this process deserves attention. PFKFB3 increases cellular lactate production by enhancing glycolysis in renal tubules. This further promotes the lactylation of histone H4 lysine 12 and enriches it at the promoters of NF-κB signaling genes such as Ikbb, Rela, and Relb, thereby activating transcription, promoting inflammatory responses, and exacerbating the progression of CKD [18]. Although this study did not explicitly demonstrate that PFKFB3-mediated inflammatory responses promote the transition from AKI to CKD, given the significant role of I/R in both AKI and CKD, it can be inferred that this mechanism may not be limited to the progression of CKD but may also be involved in the process of AKI transitioning to CKD. Furthermore, in the study of lupus nephritis (LN), the relevant research team found that due to the massive accumulation of lactate, the lactylation of transcription factor PBX1 is intensified, thereby promoting inflammation and fibrosis in LN [80]. This also provides evidence that lactylation aggravates CKD by promoting inflammation. However, it is worth noting that there is more direct evidence demonstrating the role of inflammation in the progression from AKI to CKD. Citrate synthase (CS), as a key mitochondrial enzyme, plays an important role in the TCA cycle [86]. Yu et al. found that when AKI occurs, the lactylation level of CS increases significantly in accordance with lactate concentration (particularly at the lysine residue K370). This change leads to the impairment of CS, which disrupts the TCA cycle, resulting in the accumulation of upstream metabolites and exacerbating the production of mitochondrial reactive oxygen species (mtROS). These mitochondrial stress signals act as classic triggers that subsequently drive robust activation of the NLRP3 inflammasome, thereby promoting the transition from AKI to CKD [87,88]. These findings not only unveil the underlying mechanisms by which lactylation promotes inflammation but also indicate that lactylation does not function by targeting a single molecule; rather, it serves as a key integrator of epigenetic and metabolic functions. It perpetuates the inflammatory transcriptional program through histone modifications (such as H4K12la) and simultaneously regulates inflammasome activation *via* non-histone modifications (such as CSK30), collectively driving the transition from AKI to a chronic inflammatory state and CKD.

### 5.4. Fibrosis and cell state transitions

Renal fibrosis is often a visible pathological manifestation of CKD, and the sustained progression of AKI can promote renal fibrosis, ultimately leading to the occurrence of CKD [21]. In a study on the I/R-induced AKI-CKD mouse model, researchers found that high expression of PKM2 enhances cellular glycolysis by promoting the transcription of lactate dehydrogenase A (LDHA) and glucose transporter 1 (GLUT1), thereby increasing lactate production and facilitating the transdifferentiation of pericytes into myofibroblasts. This transdifferentiation process contributes to renal interstitial fibrosis [89]. However, whether lactylation is involved in this transdifferentiation process remains to be further investigated. It is well known that unilateral ureteral obstruction (UUO) is a common cause of postrenal AKI [90]. Furthermore, UUO is an intervention used in experiments to induce CKD, so related research may also provide some indication for AKI to CKD. In a related study, it was revealed that increased lactate mediated by high PKM2 expression promotes Tgfb1 expression by stimulating lactylation of histone H3 lysine 18 in mouse renal tubular cells (TCMK-1). The released TGF-β1 further enhances M2 macrophage polarization and the Smad3 pathway-driven macrophage-myofibroblast transition (MMT) process, thereby exacerbating renal fibrosis [68]. In addition, Insulin-like growth factor binding protein 7 (IGFBP7) is commonly regarded as a biomarker for AKI. In a cadmium-induced renal fibrosis mouse model, IGFBP7 was found to promote lactate accumulation by interacting with α-enolase (ENO1) and inhibiting its ubiquitination and degradation. This lactate accumulation, in turn, enhances the transcription of IGFBP7 through lactylation of histone H3 lysine K18, thereby promoting the progression of renal fibrosis [91]. From this, we can infer that IGFBP7 may have an important influence on the progression from AKI to CKD. It is noteworthy that the transformation of macrophages into a reparative phenotype has been shown to promote the progression from AKI to CKD [8]. Experimental evidence has also shown that H4K12 lactylation in macrophages promotes their transformation into the M2 phenotype during the occurrence of CKD [66]. Accordingly, this may be an important mechanism promoting renal fibrosis.

Mitochondrial dysfunction/redox stress, metabolic reprogramming, inflammation/immune remodeling, and fibrosis/cell state transitions represent core pathological nodes driving maladaptive repair after AKI. These key processes are intricately interconnected, forming a self-perpetuating cascade that collectively propels the transition from AKI to CKD. The emerging epigenetic mechanism of lactylation not only participates individually in each of these maladaptive repair pathways but also serves as a critical molecular link that tightly couples these processes together. In the context of AKI, successful pathological repair typically requires the resolution of inflammation, characterized by normalized macrophage phenotypic switching and downregulation of inflammatory signaling. However, lactylation (such as Fis1 K20) can disrupt this process by exacerbating mitochondrial dysfunction, which in turn intensifies metabolic reprogramming [67]. This dysfunction and reprogramming further fuel persistent inflammation and fibrosis, ultimately steering the outcome toward CKD. Furthermore, lactate itself can directly amplify glycolytic flux *via* histone lactylation marks such as H3K18la and sustain the activation of the NF-κB pro-inflammatory pathway *via* H4K12la [18,70]. These mechanisms collectively contribute to the persistence of inflammation and actively impede its normal resolution. Consequently, this vicious cycle accelerates fibrotic progression and hastens the detrimental transition from AKI to CKD.

The interplay between metabolic reprogramming and epigenetic imprinting constructs a self-amplifying vicious cycle that drives the AKI-to-CKD transition. This lactate-driven ‘metabolic memory’ finds its most profound clinical expression in SAKI. Unlike transient ischemic injury, the unique feature of human sepsis is characterized by severe systemic hypoperfusion and delayed lactate clearance, resulting in an extreme and sustained ‘lactate-enriched environment.’ This, in turn, leads to a persistent landscape of lactylation in renal cells [92]. Current clinical research evidence indicates that prolonged hyperlactatemia serves as a key determinant of worsened kidney disease and poor recovery in patients with sepsis [93]. We propose that during sepsis, the intense lactate surge triggers a robust lactylation-mediated (e.g., H3K18la) ‘molecular brake,’ which confines renal tubular epithelial cells in a state of metabolic paralysis and perpetuates inflammatory responses and apoptosis [65]. This sustained epigenetic imprinting severely impedes the fundamental adaptive shift from the injury phase to proliferative repair, providing a crucial mechanistic explanation for the exceptionally high risk of progressing to irreversible CKD in SAKI patients. Given the profoundly detrimental impact of this axis in such severe clinical contexts, targeting lactate metabolism and lactylation pathways emerges as a highly promising therapeutic frontier.

## 6. Perspectives on intervention in lactylation targets to prevent the worsening of AKI

As a major risk factor for CKD, AKI provides important safeguards for preventing the occurrence of CKD through early intervention. In existing studies, it has been determined that lactylation plays a key role in the progression from AKI to CKD. Therefore, it is crucial to prevent the deterioration of AKI by intervening in lactylation targets. Since its discovery, lactylation has been proven to be involved in the onset and progression of many diseases. Particularly in the field of cancer, research on targeted intervention of lactylation sites for cancer treatment has been particularly prominent [94,95]. Given that the occurrence of lactylation is significantly positively correlated with lactate levels, consequently, the process of lactylation can be suppressed by targeting lactate metabolism-related enzymes, lactate transport proteins, as well as lactylation writer and eraser proteins.

### 6.1. Lactate metabolic enzymes

Enzymes associated with glycolysis are essential for lactate formation. Therefore, inhibiting glycolytic enzymes can effectively reduce lactate synthesis. For example, aerobic glycolysis inhibitors such as 2-deoxy-D-glucose (2-DG) and AST-120 can alleviate renal injury by inhibiting HK to reduce lactate production [70,96]. Furthermore, shikonin has been shown to exert similar effects by inhibiting PKM2, thereby alleviating renal fibrosis [68]. Moreover, cellular treatment with the LDH inhibitor GSK2837808A can also reduce lactate production, while treatment with sodium dichloroacetate (DCA) can similarly lower lactate levels in HK-2 cells by activating PDHA1 [67]. In addition to the aforementioned drugs that can affect lactate levels, a substance known as demethylzeylasteral (DML) has been demonstrated for the first time to inhibit the progression of hepatocellular carcinoma by suppressing LDH, thereby reducing the lactylation of H3K9 and H3K56 [95]. This may become a key drug for treating AKI in the future. Additionally, since these glycolytic enzyme inhibitors are not tissue- or organ-specific, it is essential to be clearly aware of their inhibitory effects on energy metabolism and their toxic effects when using these drugs.

### 6.2. Lactate transporters

It is already known that MCT is a key protein responsible for the transport of lactate into and out of cells, with MCT1 playing a dominant role in facilitating lactate uptake into cells. Therefore, inhibiting MCT1 to block lactate entry can significantly reduce intracellular lactate accumulation. MCT1-related inhibitors have been revealed to play a positive role in cancer treatment [97]. For example, AZD3965 and BAY-8002 were shown in initial studies to inhibit the growth of lymphoma by blocking lactate efflux, leading to lactate accumulation [98,99]. Although MCT1 inhibitors have shown the effect of lactate accumulation in cancer-related research, based on the properties of MCT1, it can be inferred that they may play an opposite role in other cells. In subsequent studies, MCT-related inhibitors (CHC and AZD3965) have been demonstrated to inhibit lactate and lactylation in cardiovascular diseases [100]. Therefore, we can infer that it also has a positive effect in alleviating kidney damage. However, it is noteworthy that most MCT inhibitors are currently in the preclinical stage. Moreover, given the critical physiological functions of MCT1 in various normal tissues (such as the heart, brain, etc.), developing kidney-targeted inhibitors may present a challenge to be addressed in the future.

### 6.3. Lactate writing proteins and erasing proteins

In addition to intervening in proteins related to lactate metabolism and transport, reducing lactylation levels can also be achieved by directly inhibiting lactylation writer proteins or activating eraser proteins. Garcinol, as an inhibitor of p300, can alleviate AKI by inhibiting the acetylation process [101]. As p300 also serves as a lactylation writer protein, it is worth further investigating whether garcinol can inhibit lactylation levels. Additionally, A485 and C646, as p300 inhibitors, have been demonstrated to suppress lactylation levels [102,103]. In addition to suppressing writers, the activation of erasers is also crucial for delactylation. Honokiol, as an activator of SIRT3, enhances its ability to delactylate [60]. Notably, the Free fatty acid receptor 4 (FFAR4) agonist TUG891 can alleviate cellular senescence and inhibit the progression of AKI by reversing the reduction of SIRT3 through Gq subunit-mediated CaMKKβ/AMPK signaling [104]. This may have a potential influence on the level of lactate in AKI. However, when using relevant inhibitors and activators, it is necessary to consider whether their use may lead to lactate accumulation or even lactic acidosis (Table 2).

**Table 2.** Research on intervention compound related to lactate metabolic enzymes, lactate transporters, lactate writing proteins and erasing proteins.

Compound	Action	Influence	Research on kidney	References
**Related to lactate metabolic enzymes**				
2-DG	Inhibition of glycolysis(Usually HK is suppressed)	Promote autophagy through lactate/SIRT3/AMPK pathway to prevent AKI	Yes	96
AST-120	Inhibit HK2	Inhibition of lactate production alleviates AKI, while further inhibiting HK by attenuating H3K18 lactylation	Yes	70
Shikonin	Inhibit PKM2	Reducing lactate production and H3K18 lactylation attenuates renal fibrosis and inhibits the MMT process by downregulating TGF-β1 expression	Yes	68
GSK2837808A	Inhibit LDH	Reduce lactate production, reduce 3-typ-mediated FIS1 K20 lactylation, and alleviate AKI	Yes	67
DCA	Activate PDH	Activation of PDHA1 reduces lactate production, thereby reducing FIS1 K20 lactylation and reducing AKI	Yes	67
DML	Inhibit LDH	Inhibiting lactate production to suppress H3K9 and H3K56 lactylation, thereby alleviating HCC	No	95
**Related to lactate transporters**				
AZD3965	Inhibit MCT1	Inhibition of lactate efflux leads to lactate accumulation and thus inhibits lymphoma growth	No	98
BAY-8002	Inhibit MCT1	Inhibition of lactate efflux leads to lactate accumulation and thus inhibits lymphoma growth	No	99
**Related to lactate writing proteins and erasing proteins**				
garcinol	Inhibit p300	Inhibition of acetylation to inhibit oxidative stress, inflammation and tubular cell death to inhibit AKI	Yes	101
A485	Inhibit p300	Inhibit the lactylation of YY1 K183, thereby inhibiting FGF2 transcription and then inhibiting angiogenesis	No	103
C646	Inhibit p300	Inhibit the lactylation of HMGB1	No	102
Honokiol	Activate SIRT3	Promote the delactylation of CCNE2 K348 and then inhibit HCC	No	60

2-DG: 2-Deoxy-D-Glucose; HK: Hexokinase; SIRT3: Sirtuin 3; AMPK: Adenosine Monophosphate-Activated Protein Kinase; AKI: AKI; HK2: Hexokinase 2; H3K18: Histone H3 Lysine 18; PKM2: Pyruvate Kinase M2; MMT: Mesenchymal-Myofibroblast Transition; TGF-β1: Transforming Growth Factor-Beta 1; LDH: Lactate Dehydrogenase; DCA: Dichloroacetate; PDH: Pyruvate Dehydrogenase; PDHA1: Pyruvate Dehydrogenase E1 Alpha Subunit 1; FIS1: Mitochondrial Fission 1 Protein; DML: Demethylzeylasteral; H3K9: Histone H3 Lysine 9; H3K56: Histone H3 Lysine 56; HCC: Hepatocellular Carcinoma; MCT1: Monocarboxylate Transporter 1; p300: E1A Binding Protein P300; YY1: Yin Yang 1; FGF2: Fibroblast Growth Factor 2; HMGB1: High Mobility Group Box 1; CCNE2: Cyclin E2.

## 7. Conclusion

In conclusion, the pathological progression from AKI to CKD is a complex process driven by metabolic disturbances and consolidated by epigenetic reprogramming. This review has systematically elucidated the pivotal role of the ‘lactate-lactylation’ axis, which acts as a bridge connecting acute metabolic stress to chronic tissue fibrosis and functional decline. Lactylation not only independently regulates mitochondrial homeostasis, metabolic patterns, inflammatory responses, and fibrotic processes but also serves as a core node that intertwines these maladaptive repair pathways into a difficult-to-reverse vicious cycle. Notably, apart from the risks posed by kidney disease itself, the maintenance and progression of this vicious cycle are profoundly influenced by systemic organ crosstalk, particularly the regulation of the hepato-renal metabolic axis. As the central hub for systemic lactic acid clearance, the liver acts as a crucial upstream buffer in maintaining renal homeostasis. In complex clinical settings such as sepsis or hepatorenal syndrome, the loss of this buffering function leads to systemic lactic acid overflow, subsequently establishing a persistent epigenetic ‘metabolic memory’ in the kidneys. This provides a key mechanistic explanation for the high risk of poor renal recovery observed in human SAKI patients.

However, although the broader framework of the mechanism underlying the transition from AKI to CKD has been elucidated, this field remains in its early stages, with many key details still unclear, and related research has largely been limited to the kidney as a single organ. Therefore, future research should move beyond the ‘kidney-centric’ perspective, place lactylation within a systemic framework, and more closely link fundamental molecular discoveries to clinical translation. Translating our understanding of lactylation regulation into clinical strategies—capable of effectively assessing patient risk and precisely intervening in the transition from AKI to CKD—will be the core mission and ultimate goal of future research. In summary, a deeper exploration of lactylation’s role in kidney disease not only provides a novel theoretical perspective for understanding the AKI-to-CKD transition but also opens a promising new avenue for developing innovative therapies to slow or even halt this detrimental process.

## Data Availability

Data sharing is not applicable to this article as no new data were created or analyzed in this study.
